# Processing Bodies Oscillate in Neuro 2A Cells

**DOI:** 10.3389/fncel.2019.00487

**Published:** 2019-10-29

**Authors:** Melisa Malcolm, Lucía Saad, Laura Gabriela Penazzi, Eduardo Garbarino-Pico

**Affiliations:** ^1^Departamento de Química Biológica Ranwel Caputto, Facultad de Ciencias Químicas, Universidad Nacional de Córdoba (UNC), Córdoba, Argentina; ^2^CONICET-UNC, Centro de Investigaciones en Química Biológica de Córdoba (CIQUIBIC), Córdoba, Argentina

**Keywords:** processing bodies, circadian rhythms, Neuro 2A, RNA granules, GE1/Hedls, DDX6/P54/RCK, membraneless organelles

## Abstract

Circadian rhythms are biological variables that oscillate with periods close to 24 h that are generated internally by biological clocks. Depending on the tissue/cell type, about 5–20% of genes are expressed rhythmically. Unexpectedly, the correlation between the oscillations of messengers and the proteins they encode is low. We hypothesize that these discrepancies could be because in certain phases of the circadian cycle some messengers could be translationally silenced and stored. Processing bodies (PBs) are membraneless organelles formed by ribonucleoprotein aggregates located in the cytoplasm. They contain silenced messengers and factors involved in mRNA processing. A previous work showed that the number of cells containing these mRNA granules varies when comparing two time-points in U2OS cell cultures and that these differences disappear when an essential clock gene is silenced. Here we evaluate whether PBs oscillate in Neuro2A cells. We analyzed in cell cultures synchronized with dexamethasone the variations in the number, the signal intensity of the markers used (GE-1/HEDLS and DDX6), and the area of PBs between 8 and 68 h post-synchronization. All three parameters oscillated with periods compatible with a circadian regulated process. The most robust rhythm was the number of PBs. These rhythms could be generated by oscillations in proteins that have been involved in the nucleation of these foci such as LSM1, TTP, and BRF1. The described phenomenon would allow to explain the differences observed in the temporal profiles of some messengers and their proteins and to understand how circadian clocks can control post-transcriptionally cellular functions.

## Introduction

Throughout evolution, living beings have developed mechanisms capable of measuring time and controlling numerous functions in a cyclic manner. Most of these oscillations have a period of 1 day in natural environments and close to 24 h in constant conditions. These mechanisms are called biological clocks and the functions they control circadian rhythms. They are believed to confer adaptative advantages by predicting the cyclic changes in the environment caused by the rotation of the Earth (e.g., light/dark and temperature cycles) and temporarily organizing physiology (Schibler et al., 2015; Bass and Lazar, 2016; Takahashi, 2017). Examples of circadian rhythms are cycles of sleep/wakefulness, locomotor activity or body temperature. In neurons, rhythms have been described in firing rate, gene expression, the activity of enzymes and channels and even in synaptic plasticity, among others. The molecular clockwork consists of a group of genes that mutually regulate each other through interconnected transcription-translation negative feedback loops (TTFLs) (Takahashi, 2017). These circuits rhythmically regulate the abundance of messengers encoded by ∼5–20% of genes in different tissues/organs and about 50% of mRNAs oscillate in at least some tissue (Zhang et al., 2014). Importantly, post-transcriptional regulation also plays a very important role in the generation of rhythms in protein abundance (Green, 2018). This is reflected in the poor correlation that exists between variations in the abundance of transcripts and their corresponding proteins (Reddy et al., 2006; Mauvoisin et al., 2014; Robles et al., 2014).

In the last two decades, new types of intracellular compartments characterized by not being delimited by membranes have been described in eukaryotic cells, collectively called membraneless organelles or RNA granules. They are condensed liquid-like droplets of ribonuleoprotein complexes (Courchaine et al., 2016; Sfakianos et al., 2016; Shin and Brangwynne, 2017). In cytosol, these subcompartments include processing bodies (PBs), stress granules, germ granules and neuronal granules. PBs are constitutively present, contain translationally silent mRNAs and factors involved in messengers 5′→3′ degradation, repression of translation, and RNA interference (Decker and Parker, 2012; Ivanov et al., 2018; Standart and Weil, 2018). Because of this, and other indirect evidence, it has been proposed that these foci would play a role in mRNA degradation (Sheth and Parker, 2003). However, it was also demonstrated that some messengers localizing in PBs can be translated again, consequently they would also serve as storage places (Brengues et al., 2005). Recently, it was possible to purify PBs and determine the identity of many of the proteins and mRNAs that compose them (Hubstenberger et al., 2017). From these studies it was proposed that PBs would store mRNA regulons, that is groups of transcripts coding for proteins with regulatory functions of specific pathways, while messengers with basic functions are virtually excluded (for example, transcripts coding for histone methylases but not for histones were found in PBs) (Hubstenberger et al., 2017; Standart and Weil, 2018). Thus, PBs have a role in the post-transcriptional regulation of gene expression, remarkably they would store silenced the mRNAs of groups of genes that modulate specific functions.

Since it is proposed that PBs store silenced messengers, if these structures were to oscillate, this would help to explain (at least partially) the discrepancies observed when analyzing the mRNA and protein profiles corresponding to a number of genes (Reddy et al., 2006; Mauvoisin et al., 2014; Robles et al., 2014). That is, if the messengers of a particular gene are mostly located in PBs, no matter how abundant, this will not be reflected in their protein levels or translation rate. If it is also considered that these foci contain fundamentally transcripts of regulatory factors (Hubstenberger et al., 2017; Standart and Weil, 2018), changes in their abundance and/or size would contribute to understanding the pathways by which many rhythmic cellular processes are regulated. In fact, Jang et al. (2015) have demonstrated changes in the number of cells containing PBs in cultures of U2OS cells (human osteosarcoma cell line). They decided to study this because previously, by ribosome profiling, they had found that the translation of *Lsm1* was circadian modulated into U2OS cells. LSM1 is a marker of PBs (Ingelfinger et al., 2002; Kedersha and Anderson, 2007). They also demonstrated that silencing a fundamental clock gene in the circadian clock molecular mechanism abrogates those differences (Jang et al., 2015). Given that this work analyzes only two time-points post-synchronization with dexamethasone (4 and 16 h), and that one of these is very close to the addition of this synchronizing agent that dramatically affects cellular physiology, we decided to analyze whether the number and size of PBs varies for 68 h in a neuroblastoma cell line. In this Brief Research Report we show that these two parameters oscillate cyclically in cultures of Neuro 2A cells.

## Materials and Methods

### Cell Culture

Mouse Albino neuroblastoma (Neuro 2a) cells (ATCC) were cultured in Minimum Essential Medium (MEM, Gibco) supplemented with 10% fetal bovine serum (FBS, Internegocios S.A, Argentina), in a 37°C incubator with 5% CO_2_ according ATCC recommendations. These cells have been used before for studying circadian rhythms in cell cultures (Chilov et al., 2001; Margadant et al., 2007; Repouskou et al., 2010; Chang and Guarente, 2013). Cells were grown on coverslips in a 24-well plate until they reached ∼70% of confluence, and then maintained in serum starvation conditions (0.25%, see below) to prevent the progression of the cell cycle. This is important to ensure that changes observed over time are regulated by the circadian clock and not by the cell cycle. Initially we tried to completely eliminate serum from the medium, but cells did not survive the 68 h that the experiment required. Subsequently we tested for 96 h different serum concentrations: 0, 0.1, 0.25, and 0.5%. Cells died at serum concentration lower than 0.25% and proliferate at 0.5% FBS; thus we chose 0.25% FBS, a concentration in which the number of cells did not vary during the 96 h analyzed. To control that the cells were not proliferating, we analyzed cultures grown in the same conditions by flow cytometry with propidium iodide. Indeed, when the cultures contained only 0.25% FBS in the medium, the cells were arrested. For circadian clock synchronization (i.e., to entrain the cell population to the same circadian phase), cells were treated for 1 h with 100 nM dexamethasone (Balsalobre et al., 2000; Nagoshi et al., 2005; Repouskou et al., 2010); then culture medium was replaced with fresh 0.25% FBS-MEM. Cells were fixed every 4 h for 68 h post-synchronization for immunocytochemistry analysis.

### Immunocytochemistry

Immunocytochemistry (ICC) was achieved according a protocol described by Kedersha and colleagues for analyzing PBs (Kedersha and Anderson, 2007). Briefly, Neuro 2a cells were washed twice with PBS, fixed with paraformaldehyde 4% for 15 min., permeabilized with −20°C methanol for 10 min., and incubated 1 h in blocking solution (5% Horse serum-PBS). These steps were carried out at room temperature (RT). Then cells were incubated with a mouse monoclonal antibody against p70 S6 kinase α (H-9) (Santa Cruz Biotechnology), diluted 1:1000 in blocking solution, in a humidified chamber overnight at RT. This antibody recognizes p70 S6 kinase α in the nucleus and GE-1/HEDLS/EDC4 in cytoplasm, a known marker for PBs and has been widely used for studying this foci [reviewed in 20]. Then, cells were rinsed three times with PBS, followed by incubation with a polyclonal DyLight 549-AffiniPure donkey anti-mouse IgG secondary antibody (1:2000, Jackson ImmunoResearch Labs), diluted in the same blocking solution, 30 min at RT. Nuclei were stained with Dapi. Cells were rinsed three times with PBS and mounted on slices with mowiol (Sigma-Aldrich). In addition to anti-GE-1/HEDLS, anti-DDX6/P54/RCK (Bethyl Laboratories, 1:500 in blocking solution) was used in double immunolabeling experiments. This is another recognized PB marker (Kedersha and Anderson, 2007).

### Image Detection and Analysis

Slices were visualized using an BX61 Fluorescence Microscope (Olympus) equipped with a UPLSAPO 60XO oil objective lens (NA 1.35) and U-T31 000v2 specific DAPI/Hoechs, U-TSP101para FITC, and U-T31014 Wide-band excitation set for Elphidium Bromide TRITC, Phenothrin Dil Chroma Filters. The images were acquired with a JAI^®^ CV-M4+CL monochrome camera controlled by Cytovision^®^ (Leica Biosystems). PBs were quantified according the procedure developed by Nissan and Parker (2008) for stress granules with modifications. The entire procedure was conducted using algorithms that guarantee an unbiased treatment of all pictures. Digital images of 1376 × 1038 pixels and 8 bits were processed with the ImageJ program as follow: *1)* Process menu > “Filters” > “Gaussian Blur” sigma radius 0.8 pix. *2)* Process menu > “Subtract background” (Rolling ball radius: 5 pix). *3)* Image > “Adjust” > “Threshold” 40 (red channel) or 35 (green channel). *4)* Analyze menu > “Analyze particles” > size 3–300 pix2, circularity 0.7-1. Importantly, the same threshold value was used in the analysis of all the images within each experiment, to ensure an unbiased comparison between time-points. P- bodies are considered circular, ∼300–500 nm in diameter (Cougot et al., 2012), for this reason we exclude particles without a circularity <0.7 (1 correspond to a perfect circle) or out of 3–300 pix^2^ range. This pixel size range allows quantifying particles with a diameter of 200–2000 nm. The PBs observed in Neuro 2a cells presented an average diameter of 476 nm with this procedure. We were able to decrease the upper limit to a value closer to the largest particle found, even though it would not have affected the results. P-body number was normalized by area covered by cells, for that purpose, masks were created with Fiji ImageJ, and area occupied in each microphotograph was measured.

### Statistical Analysis

Statistical analysis was performed using GraphPad Prism version 5.00 for Windows (GraphPad Software). Values are shown as mean ± SEM, unless otherwise indicated. The Kolmogorov-Smirnov’s test was used to check for normality, and Barlett’s test to check homogeneity of variances. Because non-normal distribution or homogeneity of variances were found, Kruskal–Wallis’s test was used instead of ANOVA, followed by Dunn’s multiple comparisons test; *p*-values ≤0.05 were considered as statistically significant. To evaluate periodicity in time-series data we employed MetaCycle (Wu et al., 2016), which is a R package that runs and integrates three algorithms: ARSER (Yang and Su, 2010), JTK_CYCLE (Hughes et al., 2010), and Lomb-Scargle (Glynn et al., 2006). The statistics applied with this analysis allow us to determine whether the data are periodic and, if so, their period, amplitude and phase. These parameters were applied to a Cosine function to plot the adjusted curves shown in the graph.

## Results

As mentioned in the Introduction, Jang and collaborators had shown important evidence that the number of PBs is regulated by the circadian clock (Jang et al., 2015). However, this study was limited to only two time-points, insufficient to describe a rhythm. Importantly, one of the time-points at which the number of PBs was analyzed was too close to synchronization (4 h), when the culture – and therefore the phenomenon studied – are still under the effect of the synchronizing agent (dexamethasone in this case). It is usual to start measuring at least 8 h after synchronization to analyze the rhythms independent of the effect of the agent used. We have also found, by using a database of circadian gene expression [CircaDB, Pizarro et al., 2013], that two proteins involved in the nucleation of PBs, Tristetraprolin (TTP) and BRF-1 (Franks and Lykke-Andersen, 2007), present oscillations in the levels of their messengers in murine liver (Supplementary Figures S1, S2, from CircaDB). All this together led us to analyze whether PBs show oscillations over time.

In this work we analyze whether PBs present rhythms in cultures of Neuro 2A cells, which have already been used for circadian studies (Chilov et al., 2001; Margadant et al., 2007; Repouskou et al., 2010; Chang and Guarente, 2013). These cells were established from mouse neuroblastoma, so they grow continuously and do not stop dividing under normal culture conditions (10% FBS-MEM). For this reason we first test with which serum concentration the cells stop dividing and thus prevent the progress of the cell cycle from interfering with the determinations of the analyzed variables (see section “Materials and Methods”). When the cultures reached 60–70% confluence, the cell population was synchronized with dexamethasone for 1 h and subsequently maintained at 0.25% FBS-MEM until they were fixed at the indicated time-points. PBs were detected by ICC with an antibody recognizing the GE-1/HEDLS marker. Microphotographs were taken and the PBs were quantified with ImageJ (see section “Materials and Methods”). The number of PBs showed oscillations over time (Supplementary Figure S3). The determinations were made between 8 and 68 h post-synchronization, in that lapse two peaks and two valleys can be observed in the three parameters analyzed. In Supplementary Figure S3A are shown representative pictures of these time-points (left panel) and the same photos with the mask that was obtained with the quantification procedure used (right panel). Supplementary Figure S3B shows the result of quantifying the number of PBs normalized by the area occupied by cells, the average intensity of the signal obtained after subtracting the background, and the average area of the granules at the different times analyzed. The three variables analyzed showed a similar profile. All three parameters showed significant changes over time (Kruskall–Wallis test, Supplementary Table S1 and Supplementary Tables S2–S4 show Dunn’s multiple comparison tests for the three parameters). The MetaCycle R package was used to determine whether the time series of data were rhythmic (Wu et al., 2016). This package applies statistical tests that determined that the parameters cycle with periods compatible with circadian rhythms, in addition to estimating amplitude and phase of oscillations (Supplementary Table S1). The number of PBs was the rhythm that showed the greatest amplitude. When the experiment was repeated, similar results were obtained. While the period of oscillations in signal intensity and area were comparable in the two experiments (signal intensity 24.17 vs. 22.81; area 23.34 vs. 22.46), the period of the number of PBs showed differences (30.52 vs. 23.15). In any case, taking into account that the variable is not being measured continuously and the sampling frequency (every 4 h), it is to be expected that the period cannot be determined precisely and the differences that are observed in the period estimations.

**FIGURE 1:**
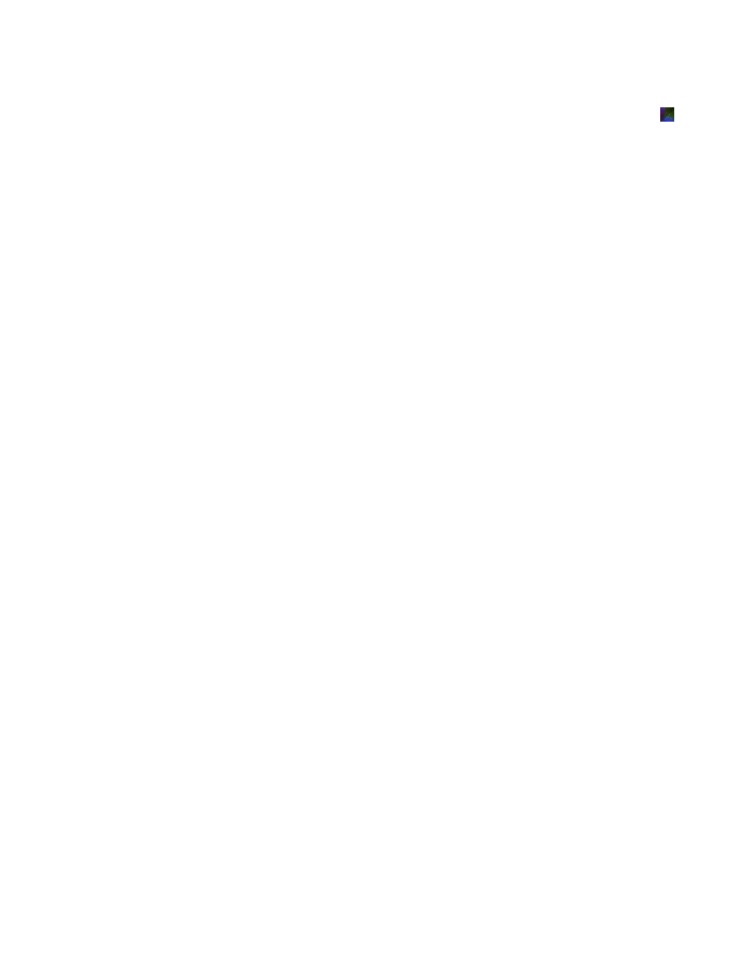
Processing Bodies oscillate cyclically in synchronized cultures of Neuro 2A cells. When the cultures reached 60–70% confluence, the medium was replaced with a fresh one containing only 0.25% serum, condition in which the cell cycle is arrested (see M&M). The following day they were synchronized with dexamethasone and fixed every 4 h between 8 and 68 h post-synchronization. The cells fixed on cover slips were treated with two antibodies by ICC, anti-GE-1/HEDLS (red) and anti-DDX6 (green), recognized PB markers. In addition, the nuclei were stained with dapi (blue). Then they were analyzed by epifluorescence microscopy and pictures were taken with a CCD camera. On the left are shown the post-synchronization time-points that are illustrated and on top the names of the markers. The images were analyzed with ImageJ and three parameters were quantified: number (per field, it was normalized considering that the field was completely covered with cells), average signal intensity and the average area of the PBs. These quantifications are shown in the right panel. The Kruskall–Wallis test followed by Dunn’s multiple comparison test was used to determine whether the changes over time were statistically significant. MetaCycle was used to assess whether the time series of data showed rhythmicity. These analyses are presented in Table 1 and Supplementary Tables S5–S10, all three parameters showed cyclic changes with the two markers. With the period, phase and amplitude values obtained by MetaCycle, the data were adjusted to a cosine-fitted curve (CFC). In the case of the PB number, the curve obtained when considering the PBs detected with the two antibodies (colocalization) is also shown.

In order to corroborate with another marker the existence of the oscillations described in PBs, we performed double immunostaining experiments analyzing DDX6 [also known as P54/RCK, another recognized PB marker (Kedersha and Anderson, 2007)] together with GE-1/HEDLS. The cultures were synchronized and the same time-points were taken as in Supplementary Figure S1, although in this case two antibodies were used in the ICC. Figure 1 and Table 1 show that the number, signal intensity and area of PBs showed relatively similar profiles with both markers. All three variables, with both antibodies, showed statistically significant changes over time in the form of periodic oscillations of at least two cycles (Kruskall–Wallis test and MetaCycle, Table 1; Supplementary Tables S5–S10, Dunn’s multiple comparison tests. *n* = 16–24 pictures per time-point). In each microphotography there were 23.6 ± 9.1 nuclei (average ± SD); that is, between 378 and 566 cells were analyzed at each time-point. Remarkably, all three parameters showed higher levels in the second cycle when PBs were detected with anti-DDX6 (Figure 1 and Table 1). The PB number was the variable that showed the greatest amplitude with the two markers. It was normalized by the surface occupied by the cells since several of them were not completely included in the pictures. Considering this, when the PB number was divided by the number of nuclei, on average this coefficient was between 2.3 and 7.9 per cell when GE-1/HEDLS was used and between 2.7 and 10.8 with DDX6. The period estimated with the two antibodies was very similar, 23.57 h with anti-GE-1/HEDLS and 23.33 h with anti-DDX6. When only granules marked with both markers were considered, as expected the number decreased. Even so the behavior over time remained rhythmic (Fig. 1. *p* = 2.19E-11 by MetaCycle). When the signal intensity was analyzed, the overall values were different with each antibody, this is to be expected since they are different antibodies and fluorophores, although the time pattern was relatively similar but with greater amplitude for GE-1/HEDLS (Figure 1 and Table 1). In the case of the PB area, as anticipated, the values were reduced since in general the overlap of the signal was not complete, which means that not all the pixels recognized with one antibody as a PB matched exactly with those detected by the other. However, the temporal behavior also showed to be statistically rhythmic (*p* = 5.3E-4). This experiment was repeated and showed similar results; therefore, these oscillations were demonstrated in 4 independent experiments with anti-GE-1/HEDLS and in 2 with anti-DDX6. In these repetitions minor changes in the phase and amplitude of the rhythms are observed, this is expected since the measurements were not made in a continuous way but with intervals of 4 h and taking different samples in each time-point; nevertheless the temporal profiles are similar in all the cases.

**TABLE 1:** PB temporal oscillations in Neuro 2a cells. PB number (normalized per field).

**DDX6**			**GE-1/HEDLS**			**DDX6**			**GE-1/HEDLS**		
**t(h)**	**Mean**	**SEM**	**Mean**	**SEM**	***n***	**t(h)**	**Mean**	**SEM**	**Mean**	**SEM**	***n***
8	158.50	78.03	233.90	61.24	20	40	440.14	440.14	243.39	117.03	20
12	135.88	55.14	193.19	57.38	24	44	441.96	441.96	303.24	91.81	20
16	107.21	44.95	149.42	71.50	18	48	458.20	458.20	328.19	89.84	20
20	228.78	95.91	222.16	77.44	21	52	343.16	343.16	257.67	86.02	16
24	188.79	77.82	215.81	66.44	20	56	436.80	436.80	265.86	102.40	20
28	326.62	125.14	518.41	129.08	19	60	330.72	330.72	194.10	66.05	25
32	322.07	93.82	334.35	150.35	20	64	318.47	318.47	159.11	69.52	20
36	217.18	142.59	152.55	82.95	19	68	399.98	399.98	260.99	73.01	17

**Statistical analysis**		**Kruskall-Wallis DDX6**						**Kruskall-Wallis GE-1/HEDLS**			
									
			***P***	**H**					***P***	**H**	
									
			P < 0.0001	171.50					P < 0.0001	132.30	
					
		**MetaCycle (meta2d)1 DDX6**						**MetaCycle (meta2d)1GE-1/HEDLS**			
		***P***	**Period**	**Phase**	**Amp**			***P***	**Period**	**Phase**	**Amp**
					
		5.32E-04	23.33	1.17	41.12			0.00	23.57	647	57.79

**Signal intensity (a.u.)**											

**DDX6**			**GE-1/HEDLS**			**DDX6**			**GE-1/HEDLS**		
**t(h)**	**Mean**	**SEM**	**Mean**	**SEM**	***n***	**t(h)**	**Mean**	**SEM**	**Mean**	**SEM**	***n***
8	39.48	2.18	57.93	3.20	20	40	45.56	2.60	58.90	3.59	20
12	39.61	2.02	58.50	2.62	24	44	46.41	1.73	61.00	2.15	20
16	37.94	1.63	53.21	2.19	15	48	46.49	2.02	60.72	1.91	20
20	41.27	2.86	57.84	3.83	21	52	45.19	1.78	59.37	2.54	16
24	39.75	1.70	56.28	2.01	20	56	46.64	3.46	59.72	3.96	20
28	40.08	1.66	59.68	2.63	19	60	46.97	2.78	60.23	3.12	25
32	41.83	1.52	58.10	2.02	20	64	47.14	3.13	59.63	4.29	20
36	40.15	2.19	55.31	2.76	19	68	47.31	2.28	62.19	3.42	17

**Satistical analysis**		**Kruskall-Wallis DDX6**						**Kruskall-Wallis GE-1/HEDLS**			
									
			***P***	**H**					***P***	**H**	
									
			P < 0.0001	232.70					P < 0.0001	113.30	
					
		**MetaCycle (meta2d)1 DDX6**						**MetaCycle (meta2d)1 GE-1/HEDLS**			
					
		***P***	**Period**	**Phase**	**Amp**			***P***	**Period**	**Phase**	**Amp**
					
		1.85E-06	20.60	0.44	0.44			341E-07	19.93	7.22	1.34

**Area (pixel^2^)**											

**DDX6**			**GE-1/HEDLS**			**DDX6**			**GE-1/HEDLS**		
**t(h)**	**Mean**	**SEM**	**Mean**	**SEM**	***n***	**t(h)**	**Mean**	**SEM**	**Mean**	**SEM**	***n***

8	10.70	2.36	16.12	2.38	20	40	14.95	2.41	16.56	2.81	20
12	11.80	2.02	16.72	1.75	24	44	16.19	1.84	17.97	1.83	20
16	10.37	2.15	13.41	1.82	15	48	15.25	1.86	17.88	1.80	20
20	12.39	2.44	16.28	2.73	21	52	13.89	1.65	16.67	1.84	16
24	10.97	1.30	14.60	1.74	20	56	16.07	3.37	17.53	3.46	20
25	11.19	1.81	16.98	1.90	19	60	15.39	2.47	17.35	2.38	25
32	12.25	1.27	15.78	1.56	20	64	16.07	2.79	17.90	3.19	20
36	11.07	1.92	14.21	1.74	19	68	16.05	1.90	18.63	2.73	17

**Satistical analysis**		**Kruskall-Wallis DDX6**						**Kruskall-Wallis GE-1/HEDLS**			
									
			***P***	**H**					***P***	**H**	
									
			*P* < 0.0001	182.50					*P* < 0.0001	91.06	
					
		**MetaCycle (meta2d)1 DDX6**						**MetaCycle (meta2d)1 GE-1/HEDLS**			
		***P***	**Period**	**Phase**	**Amp**			***p***	**Period**	**Phase**	**Amp**
					
		141E-06	22.74	20.52	144			0.00	19.72	7.87	0.80

*Note that the mean was calculated as the mean of the means of each micmphotograph. N indicates the number of microphotographs per time analyzed. Each picture contain an average of 23 cells per field.*

## Conclusion

This work shows for the first time oscillations in the dynamics of PBs. These rhythms were evidenced both in the number, intensity levels of the marker used, and the area of these cytoplasmic ribonucleoprotein granules with two different markers. A previous study had shown differences in the number of PBs when comparing two time-points in U2OS cells, as well as that this difference disappeared by silencing a clock gene essential for the functioning of the circadian clock molecular mechanism (Jang et al., 2015). Taken together, these works indicate that PBs are modulated by circadian clocks. Interestingly, both we (manuscript submitted) and another group (Wang et al., 2019), have found that other type of mRNA granules, the stress granules, also have temporal variations in their number.

In this brief report we limit ourselves to presenting the phenomenon, we have not explored the mechanisms by which the rhythms described in PBs are generated. A plausible hypothesis is that the circadian clock control the levels of factors that can induce the formation of PBs. In fact, changes in the rate of translation of a marker of these foci, LSM1, suggested to Jang and colleagues that PBs could be controlled by the circadian clock (Jang et al., 2015). We searched the CircaDB database (Pizarro et al., 2013) for messengers of two other of these proteins, TTP and BRF1 (Franks and Lykke-Andersen, 2007), that oscillated in their levels, and in fact do so (Supplementary Figures S1, S2). Other proteins that induce the formation of PBs may also participate in the generation of the observed rhythms. In addition to changes in the concentration of these proteins, post-translational modifications that induce the phase transitions that form PBs have also been described. If any of these modifications are rhythmically controlled, this could also contribute to the phenomenon described.

It is currently postulated that PBs are membrane-free compartments where transcripts are stored (Hubstenberger et al., 2017; Standart and Weil, 2018). They have also been involved in translation silencing and mRNA degradation [discussed in Decker and Parker (2012), Ivanov et al. (2018)]. Since at the beginning of the century technologies were available to analyze the transcriptome and the proteome globally, circadian experiments were performed that demonstrated a significant number of genes (∼50%) showed, contrary to expectations, a poor correlation between the abundance rhythms of their mRNA and protein (Reddy et al., 2006; Mauvoisin et al., 2014; Robles et al., 2014). The presence of many of these messengers in PBs, instead of in polysomes, could explain that even being abundant, their corresponding proteins would not be present in the same magnitude.

Considering the evidence suggesting that PBs store groups of messengers that participate in the regulation of specific pathways [mRNA regulons (Hubstenberger et al., 2017)], the fact that these foci are rhythmically regulated would contribute to understand how clocks can control a number of circadian rhythms at a post-transcriptional level.

## Data Availability Statement

All datasets generated for this study are included in the article/Supplementary Material.

## Author Contributions

MM, LS, and LP: acquisition, analysis and interpretation of data, making of the figures, revising the work, and design critically. EG-P: conception and design of the work, and manuscript writing. All authors contributed to the manuscript revision, and read and approved the submitted version.

## Conflict of Interest

The authors declare that the research was conducted in the absence of any commercial or financial relationships that could be construed as a potential conflict of interest.
