# Supplementary material for: Processing Bodies Oscillate in Neuro 2A Cells
Source: Front Cell Neurosci. 2019 Oct 29;13:487. doi: 10.3389/fncel.2019.00487 (PMC6828937; doi:10.3389/fncel.2019.00487)
Supplement: Supplementary file 1 [file Data_Sheet_1.PDF]

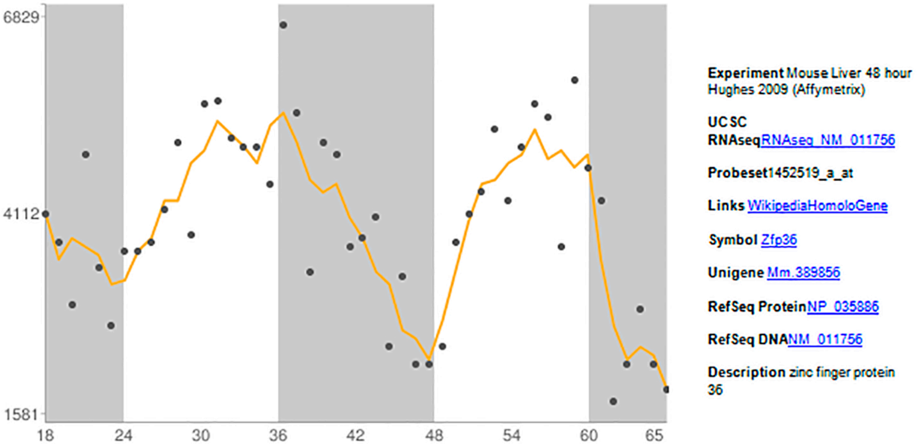

	p-Value	q-Value	period	phase
JTK	1.62e-05	0.000408965	21.5	9.75
Lomb Scargle	0.000267	0.00814197	20.945	17.02
DeLichtenberg	0.0297	0.568452	24.0	*NA*

Supplementary Figure 1: *TTP/Zfp36* mRNA oscilations in mouse liver. From CircaDB database (http://circadb.hogeneschlab.org)
