# Supplementary material for: Processing Bodies Oscillate in Neuro 2A Cells
Source: Front Cell Neurosci. 2019 Oct 29;13:487. doi: 10.3389/fncel.2019.00487 (PMC6828937; doi:10.3389/fncel.2019.00487)
Supplement: Supplementary file 2 [file Data_Sheet_2.PDF]

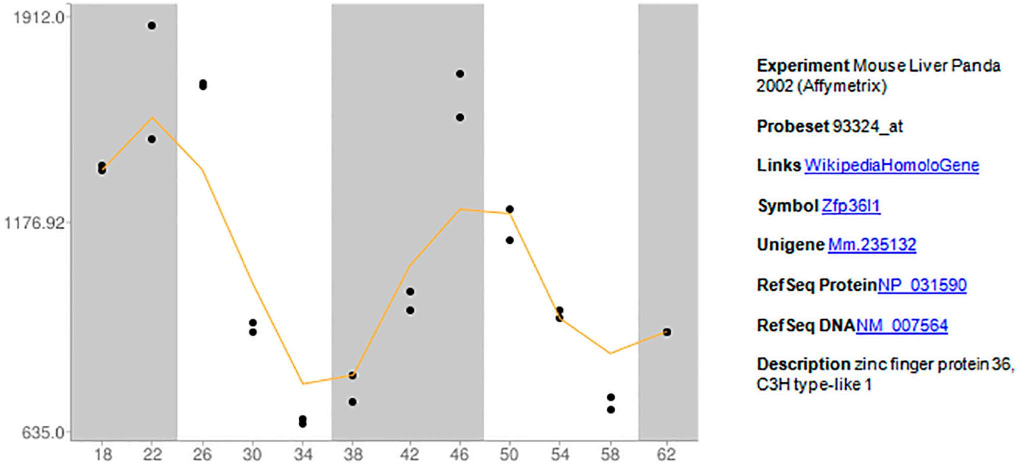

	p-Value	q-Value	period	phase
JTK	1.8e-08	3.0e-05	24.0	0.0
Lomb Scargle	0.00241	1.0	29.578	1.75
DeLichtenberg	0.7137	1.0	24.0	*NA*

Supplementary Figure 2: *Brf1/Zfp36l1* mRNA oscilations in mouse liver. From CircaDB database (http://circadb.hogeneschlab.org)
