# Supplementary material for: Processing Bodies Oscillate in Neuro 2A Cells
Source: Front Cell Neurosci. 2019 Oct 29;13:487. doi: 10.3389/fncel.2019.00487 (PMC6828937; doi:10.3389/fncel.2019.00487)
Supplement: Supplementary file 3 [file Data_Sheet_3.PDF]

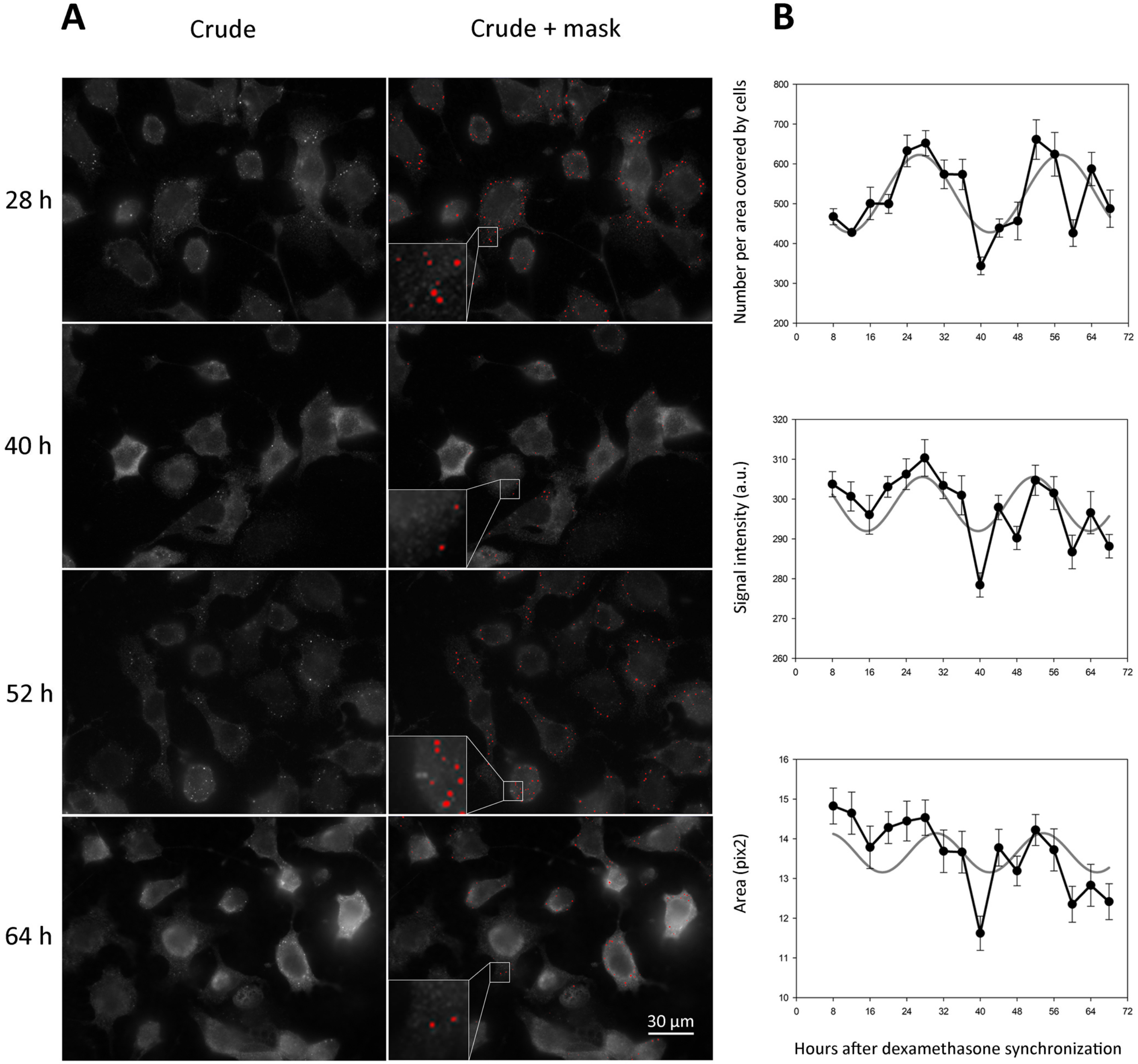

<u>Suppl. figure 3</u>: Processing Bodies oscillate cyclically in synchronized cultures of Neuro 2A cells. When the cultures reached 60-70% confluence, the medium was replaced with a fresh one containing only 0.25% serum, condition in which the cell cycle is arrested (see M&M). The following day they were synchronized with dexamethasone and fixed every 4 h between 8 and 68 h post-synchronization. The cells fixed on cover slips were treated by ICC with an anti-GE-1/HEDLS antibody. Then they were analyzed by epifluorescence microscopy and pictures were taken with a CCD camera. In the left panel pictures taken from samples corresponding to the indicated time-points are shown. In each case the microphotographs corresponding to the marker and the same picture with the quantification mask are shown. The images were analyzed with ImageJ and three parameters were quatified: number (per field, it was normalized considering that the field was completely covered with cells), average signal intensity and the average area of the PBs. These quantifications are shown in the right panel. The Kruskall-Wallis test followed by Dunn´s multiple comparison test was used to determine whether the changes over time were statistically significant. MetaCycle was used to assess whether the time series of data showed rhythmicity. These analyses are presented in Suppl. tables 1-4, all three parameters showed cyclic changes. With the period, phase and amplitude values obtained by MetaCycle, the data were adjusted to a cosine-fitted curve (CFC).
