# Supplementary material for: Processing Bodies Oscillate in Neuro 2A Cells
Source: Front Cell Neurosci. 2019 Oct 29;13:487. doi: 10.3389/fncel.2019.00487 (PMC6828937; doi:10.3389/fncel.2019.00487)
Supplement: Supplementary file 4 [file Data_Sheet_4.PDF]

Suppl. Table 1: Processing body temporal oscillations in Neuro 2a cells.

a) *Processing bodies per area covered by cells*

t (h)	Mean	SEM	Min.	Max.	n	t (h)	Mean	SEM	Min.	Max.	n
8	467.35	20.34	292.75	555.85	15	40	343.91	22.10	203.36	495.24	15
12	427.64	15.75	303.84	551.45	15	44	438.73	22.92	286.67	548.34	15
16	500.72	40.88	275.64	842.83	15	48	456.58	47.46	260.87	857.61	15
20	499.74	23.70	337.62	696.97	15	52	661.33	49.70	266.23	1009.91	15
24	632.71	39.56	461.87	1012.71	15	56	624.31	54.90	131.56	918.25	15
28	652.09	31.44	448.68	901.41	16	60	426.24	33.32	301.13	756.23	15
32	574.04	35.86	366.04	841.53	15	64	587.36	41.91	305.16	925.17	15
36	573.59	38.03	340.98	857.45	15	68	487.69	46.91	231.60	813.42	15

Statistical analysis	Kruskall-Wallis		MetaCycle (meta2d)			
	p	H	p	Period	Phase	Amp
	<0.0001	78.73	4.81E-14	30.52	26.70	97.40

b) *Signal intensity (GE-1/HEDLS fluorescent immunosignal)*

t (h)	Mean	SEM	Min.	Max.	n	t (h)	Mean	SEM	Min.	Max.	n
8	303.75	3.17	277.34	321.19	15	40	278.42	3.04	260.60	306.24	15
12	300.68	3.65	273.80	312.19	15	44	297.92	3.08	279.23	319.13	15
16	296.06	4.88	266.82	336.49	15	48	290.24	2.91	274.09	310.28	15
20	303.08	2.61	286.39	322.35	15	52	304.73	3.80	278.36	329.10	15
24	306.26	3.86	284.11	329.58	15	56	301.51	4.13	275.93	328.91	15
28	310.34	4.60	11.83	18.20	16	60	286.72	4.24	263.89	322.33	15
32	303.41	3.29	284.74	335.83	15	64	296.58	5.30	282.50	353.49	15
36	300.95	4.91	277.67	344.52	15	68	288.15	2.96	283.41	310.26	15

Statistical analysis	Kruskall-Wallis		MetaCycle (meta2d)			
	p	H	p	Period	Phase	Amp
	<0.0001	61.71	1.48E-06	24.17	3.32	6.80

c) *Area (pixels^2)*

t (h)	Mean	SEM	Min.	Max.	n	t (h)	Mean	SEM	Min.	Max.	n
8	14.82	0.45	11.85	17.38	15	40	11.62	0.43	10.49	16.36	15
12	14.64	0.53	10.00	17.99	15	44	13.77	0.46	10.69	17.23	15
16	13.79	0.53	10.17	17.66	15	48	13.19	0.37	10.64	15.79	15
20	14.28	0.40	12.15	17.27	15	52	14.22	0.39	11.85	17.16	15
24	14.45	0.50	10.79	18.70	15	56	13.72	0.53	11.12	19.15	15
28	14.53	0.44	11.83	18.20	16	60	12.35	0.45	9.36	16.32	15
32	13.69	0.54	11.32	18.79	15	64	12.83	0.53	10.82	17.74	15
36	13.66	0.53	10.73	17.78	15	68	12.41	0.45	8.36	15.32	15

Statistical analysis	Kruskall-Wallis		MetaCycle (meta2d)			
	p	H	p	Period	Phase	Amp
	<0.0001	50.83	2.29E-03	23.34	7.03	0.49

***** *Note that the mean was calculated as the mean of the means of each microphotograph; minimum and maximum values correspond to the mean of the photos with the lowest and highest mean at each time-point, respectively."n" is the number of pictures analyzed at each time-point.*
