# Supplementary material for: Processing Bodies Oscillate in Neuro 2A Cells
Source: Front Cell Neurosci. 2019 Oct 29;13:487. doi: 10.3389/fncel.2019.00487 (PMC6828937; doi:10.3389/fncel.2019.00487)
Supplement: Supplementary file 5 [file Data_Sheet_5.PDF]

Suppl. Table 2: Processing body number per area covered by cells (GE-1/HEDLS).

T (h)	8	12	16	20	24	28	32	36	40	44	48	52	56	60	64	68
**8**																
**12**	24.80															
**16**	-9.87	-34.67														
**20**	-15.73	-40.53	-5.87													
**24**	-68.07	**-92.87***	-58.20	-52.33												
**28**	-78.69	**-103.50****	-68.83	-62.96	-10.63											
**32**	-44.73	-69.53	-34.87	-29.00	23.33	33.96										
**36**	-45.40	-70.20	-35.53	-29.67	22.67	33.29	-0.67									
**40**	59.87	35.07	69.73	75.60	**127.90*****	**138.60*****	**104.60****	**105.30****								
**44**	12.93	-11.87	22.80	28.67	81.00	**91.63***	57.67	58.33	-46.93							
**48**	12.27	-12.53	22.13	28.00	80.33	**90.96***	57.00	57.67	-47.60	-0.67						
**52**	-71.87	**-96.67***	-62.00	-56.13	-3.80	6.83	-27.13	-26.47	**-131.70*****	-84.80	-84.13					
**56**	-60.73	-85.53	-50.87	-45.00	7.33	17.96	-16.00	-15.33	**-120.60*****	-73.67	-73.00	11.13				
**60**	24.00	-0.80	33.87	39.73	**92.07***	**102.70****	68.73	69.40	-35.87	11.07	11.73	**95.87***	84.73			
**64**	-48.27	-73.07	-38.40	-32.53	19.80	30.43	-3.53	-2.87	**-108.10****	-61.20	-60.53	23.60	12.47	-72.27		
**68**	-7.67	-32.47	2.20	8.07	60.40	71.03	37.07	37.73	-67.53	-20.60	-19.93	64.20	53.07	-31.67	40.60	

Dunn´s Multiple Comparison test for variable Processing bodies covered by cells. Difference in rank sum.

***In bold p ≤ 0.05.**
