# Supplementary material for: Processing Bodies Oscillate in Neuro 2A Cells
Source: Front Cell Neurosci. 2019 Oct 29;13:487. doi: 10.3389/fncel.2019.00487 (PMC6828937; doi:10.3389/fncel.2019.00487)
Supplement: Supplementary file 6 [file Data_Sheet_6.PDF]

Suppl. Table 3: Processing body Signal Intensity (GE-1/HEDLS marker).

T (h)	8	12	16	20	24	28	32	36	40	44	48	52	56	60	64	68
**8**																
**12**	14.87															
**16**	40.47	25.60														
**20**	4.20	-10.67	-36.27													
**24**	-5.07	-19.93	-45.53	-9.27												
**28**	-15.13	-30.00	-55.60	-19.33	-10.07											
**32**	5.87	-9.00	-34.60	1.67	10.93	21.00										
**36**	26.53	11.67	-13.93	22.33	31.60	41.67	20.67									
**40**	**113.10****	**98.20***	72.60	**108.90****	**118.10*****	**128.20*****	**107.20****	86.53								
**44**	29.47	14.60	-11.00	25.27	34.53	44.60	23.60	2.93	-83.60							
**48**	66.13	51.27	25.67	61.93	71.20	81.27	60.27	39.60	-46.93	36.67						
**52**	-2.27	-17.13	-42.73	-6.47	2.80	12.87	-8.13	-28.80	**-115.30*****	-31.73	-68.40					
**56**	15.93	1.07	-24.53	11.73	21.00	31.07	10.07	-10.60	**-97.13***	-13.53	-50.20	18.20				
**60**	77.87	63.00	37.40	73.67	82.93	**93.00***	72.00	51.33	-35.20	48.40	11.73	80.13	61.93			
**64**	51.13	36.27	10.67	46.93	56.20	66.27	45.27	24.60	-61.93	21.67	-15.00	53.40	35.20	-26.73		
**68**	73.87	59.00	33.40	69.67	78.93	**89.00***	68.00	47.33	-39.20	44.40	7.73	76.13	57.93	-4.00	22.73	

Dunn´s Multiple Comparison test for variable Signal intensity. Difference in rank sum

*** In bold p ≤ 0.05.**
