# Supplementary material for: Processing Bodies Oscillate in Neuro 2A Cells
Source: Front Cell Neurosci. 2019 Oct 29;13:487. doi: 10.3389/fncel.2019.00487 (PMC6828937; doi:10.3389/fncel.2019.00487)
Supplement: Supplementary file 7 [file Data_Sheet_7.PDF]

Suppl. Table 4: Processing body Area (GE-1/HEDLS marker).

T (h)	8	12	16	20	24	28	32	36	40	44	48	52	56	60	64	68
**8**																
**12**	5.47															
**16**	36.07	30.60														
**20**	16.73	11.27	-19.33													
**24**	11.07	5.60	-25.00	-5.67												
**28**	10.47	5.00	-25.60	-6.27	-0.60											
**32**	44.60	39.13	8.53	27.87	33.53	34.13										
**36**	42.80	37.33	6.73	26.07	31.73	32.33	-1.80									
**40**	**114.90*** **	**109.40** **	78.80	**98.13***	**103.80** **	**104.40** **	70.27	72.07								
**44**	34.07	28.60	-2.00	17.33	23.00	23.60	-10.53	-8.73	-80.80							
**48**	54.47	49.00	18.40	37.73	43.40	44.00	9.87	11.67	-60.40	20.40						
**52**	18.40	12.93	-17.67	1.67	7.33	7.93	-26.20	-24.40	**-96.47***	-15.67	-36.07					
**56**	42.80	37.33	6.73	26.07	31.73	32.33	-1.80	0.00	-72.07	8.73	-11.67	24.40				
**60**	87.87	82.40	51.80	71.13	76.80	77.40	43.27	45.07	-27.00	53.80	33.40	69.47	45.07			
**64**	78.13	72.67	42.07	61.40	67.07	67.66	33.53	35.33	-36.73	44.07	23.67	59.73	35.33	-9.73		
**68**	76.30	70.83	40.23	59.57	65.23	65.83	31.70	33.50	-38.57	42.23	21.83	57.90	33.50	-11.57	-1.83	

Dunn´s Multiple Comparison test for variable Area. Difference in rank sum.

*** In bold p ≤ 0.05.**
