# Supplementary material for: Processing Bodies Oscillate in Neuro 2A Cells
Source: Front Cell Neurosci. 2019 Oct 29;13:487. doi: 10.3389/fncel.2019.00487 (PMC6828937; doi:10.3389/fncel.2019.00487)
Supplement: Supplementary file 8 [file Data_Sheet_8.PDF]

Suppl. Table 5: Processing body number per area covered by cells. (GE-1/HEDLS marker in Fig.1).

T (h)	8	12	16	20	24	28	32	36	40	44	48	52	56	60	64	68
**8**																
**12**	44.05															
**16**	85.47	41.42														
**20**	15.68	-28.37	-69.79													
**24**	18.95	-25.10	-66.52	3.27												
**28**	**-143.80*** **	**-187.80*** **	**-229.20*** **	**-159.40*** **	**-162.70*** **											
**32**	-63.27	**-107.30***	**-148.70*** **	-78.95	-82.22	80.48										
**36**	87.35	43.30	1.89	71.67	68.40	**231.10*** **	**150.60*** **									
**40**	-0.46	-44.51	-85.93	-16.14	-19.41	**143.30*** **	62.81	-87.81								
**44**	-58.55	**-102.60***	**-144.00*** **	-74.23	-77.50	85.20	4.72	**-145.90*** **	-58.09							
**48**	-75.61	**-119.70*** **	**-161.10*** **	-91.29	-94.56	68.14	-12.34	**-163.00*** **	-75.15	-17.06						
**52**	-21.39	-65.44	-106.90	-37.07	-40.34	**122.40***	41.88	-108.70	-20.93	37.16	54.23					
**56**	-22.20	-66.25	**-107.70***	-37.88	-41.15	**121.60****	41.07	**-109.60***	-21.74	36.35	53.41	-0.81				
**60**	40.78	-3.27	-44.69	25.10	21.83	**184.50*** **	**104.10***	-46.57	41.24	99.33	**116.40*** **	62.17	62.98			
**64**	78.00	33.95	-7.47	62.32	59.05	**221.80*** **	**141.30*** **	-9.35	78.46	**136.60*** **	**153.60*** **	99.39	100.20	37.22		
**68**	-26.88	-70.93	-112.30	**-42.56**	-45.83	**116.90***	36.39	**-114.20***	-26.42	31.67	48.74	-5.49	-4.68	-67.66	-104.9	

Dunn´s Multiple Comparison test for variable Processing bodies covered by cells. Difference in rank sum.
*In bold p ≤ 0.05.
