# Supplementary material for: Processing Bodies Oscillate in Neuro 2A Cells
Source: Front Cell Neurosci. 2019 Oct 29;13:487. doi: 10.3389/fncel.2019.00487 (PMC6828937; doi:10.3389/fncel.2019.00487)
Supplement: Supplementary file 9 [file Data_Sheet_9.PDF]

Suppl. Table 6: Processing body Signal Intensity (GE-1/HEDLS marker in Fig. 1).

T (h)	8	12	16	20	24	28	32	36	40	44	48	52	56	60	64	68
8																
12	-15.45															
16	108.80	124.3**														
20	6.47	21.92	-167.70***													
24	47.35	62.80	-116.30*	40.88												
28	-58.92	-43.47	-41.89	-65.39	-106.30											
32	-7.48	7.97	-144.60***	-13.95	-54.83	51.44										
36	66.92	82.37	-202.40***	60.45	19.57	125.80**	74.40									
40	-35.77	-20.32	-196.80***	-42.24	-83.12	23.15	-28.29	-102.70*								
44	-93.55	-78.10	-152.40***	-100.00	-140.90***	-34.63	-86.07	-160.50***	-57.78							
48	-87.96	-72.52	-153.70***	-94.44	-135.30***	-29.04	-80.48	-154.90***	-52.19	5.59						
52	-43.61	-28.17	-178.60***	-50.09	-90.96	15.31	-36.13	-110.50	-7.84	49.94	44.35					
56	-44.87	-29.42	-151.40***	-51.34	-92.22	14.05	-37.39	-111.80*	-9.10	48.68	43.09	-1.26				
60	-69.75	-54.30	-218.40***	-76.22	-117.10**	-10.83	-62.27	-136.70***	-33.98	23.80	18.21	-26.14	-24.88			
64	-42.60	-27.15	-167.70***	-49.07	-89.95	16.32	-35.12	-109.50*	-6.83	50.95	45.36	1.01	2.27	27.15		
68	-109.60	-94.16	-116.30***	-116.10*	-157.00***	-50.69	-102.10	-176.50***	-73.84	-16.06	-21.65	-66.00	-64.74	-39.86	-67.01	

Dunn´s Multiple Comparison test for variable Processing bodies covered by cells. Difference in rank sum.
*In bold $p \leq 0.05$.
