# Supplementary material for: Processing Bodies Oscillate in Neuro 2A Cells
Source: Front Cell Neurosci. 2019 Oct 29;13:487. doi: 10.3389/fncel.2019.00487 (PMC6828937; doi:10.3389/fncel.2019.00487)
Supplement: Supplementary file 10 [file Data_Sheet_10.pdf]

Suppl. Table 7: Processing body Area (GE-1/HEDLS marker in Fig. 1).

T (h)	8	12	16	20	24	28	32	36	40	44	48	52	56	60	64	68
**8**																
**12**	-24.83															
**16**	97.33	**122.20****														
**20**	1.76	26.60	-95.57													
**24**	57.63	82.46	-39.71	55.86												
**28**	-37.13	-12.30	**-134.50****	-38.89	-94.76											
**32**	10.55	35.38	-86.79	8.78	-47.08	47.68										
**36**	72.95	97.78	-24.39	71.19	15.32	**110.10***	62.40									
**40**	-19.22	5.61	**-116.60****	-20.98	-76.85	17.91	-29.77	-92.17								
**44**	-75.75	-50.92	**-173.10*****	-77.51	**-133.40****	-38.62	-86.30	**-148.70**	-56.53							
**48**	-73.59	-48.75	**-170.90*****	-75.35	**-131.20****	-36.46	-84.13	**-146.50**	-54.37	2.16						
**52**	-24.41	0.43	**-121.70***	-26.17	-82.03	12.73	-34.95	-97.35	-5.18	51.34	49.18					
**56**	-40.02	-15.19	**-137.40*****	-41.78	-97.65	-2.89	-50.57	**-113.00**	-20.80	35.73	33.56	-15.62				
**60**	-45.46	-20.63	**-142.80*****	-47.22	**-103.10***	-8.33	-56.01	**-118.40**	-26.24	30.29	28.13	-21.05	-5.44			
**64**	-55.05	-30.22	**-152.40*****	-56.81	**-112.70***	-17.92	-65.60	**-128.00**	-35.83	20.70	18.54	-30.64	-15.03	-9.59		
**68**	-88.00	-63.17	**-185.30*****	-89.76	**-145.60*****	-50.87	-98.55	**-160.90**	-68.78	-12.25	-14.41	-63.59	-47.98	-42.54	-32,95	

Dunn´s Multiple Comparison test for variable Processing bodies covered by cells. Difference in rank sum.
*In bold $p \leq 0.05$.
