# Supplementary material for: Processing Bodies Oscillate in Neuro 2A Cells
Source: Front Cell Neurosci. 2019 Oct 29;13:487. doi: 10.3389/fncel.2019.00487 (PMC6828937; doi:10.3389/fncel.2019.00487)
Supplement: Supplementary file 11 [file Data_Sheet_11.PDF]

Suppl. Table 8: Processing body number per area covered by cells (DDX6 marker in Fig. 1).

T (h)	8	12	16	20	24	28	32	36	40	44	48	52	56	60	64	68
**8**																
**12**	17.36															
**16**	36.84	19.49														
**20**	-50.62	-67.98	-87.47													
**24**	-20.25	-37.61	-57.09	30.37												
**28**	**-110.90***	**-128.30**	**-147.80*****	-60.32	-90.69											
**32**	**-114.90***	**-132.20**	**-151.70*****	-64.24	-94.61	-3.92										
**36**	-37.05	-54.41	-73.89	13.58	-16.80	73.89	77.81									
**40**	**-161.80*****	**-179.10****	**-198.60*****	**-111.20***	**-141.50*****	-50.84	-46.92	**-124.70****								
**44**	**-163.20*****	**-180.50*****	**-200.00*****	**-112.50***	**-142.90*****	-52.21	-48.29	**-126.10****	-1.37							
**48**	**-181.10*****	**-198.50*****	**-218.00*****	**-130.50*****	**-160.90*****	-70.20	-66.28	**-144.10*****	-19.36	-17.99						
**52**	**-117.20***	**-134.50****	**-154.00*****	-66.54	-96.91	-6.22	-2.30	-80.12	44.62	45.99	63.98					
**56**	**-156.30*****	**-173.60*****	**-193.10*****	**-105.70***	-136.00	-45.34	-41.42	**-119.20****	5.50	6.87	24.86	-39.12				
**60**	**-109.20***	**-126.60*****	**-146.10*****	-58.60	-88.97	1.72	5.64	-72.17	52.56	53.93	71.92	7.94	47.06			
**64**	-103.70	**-121.00****	**-140.50*****	-53.03	-83.40	7.29	11.21	-66.60	58.13	59.50	77.49	13.51	52.63	5.57		
**68**	**-158.60*****	**-175.90*****	**-195.40*****	-107.90	**-138.30****	-47.63	-43.71	**-121.50***	3.21	4.58	22.57	-41.41	-2.29	-49.35	-54.92	

Dunn´s Multiple Comparison test for variable Processing bodies covered by cells. Difference in rank sum.
*In bold $p \leq 0.05$.
