# Supplementary material for: Processing Bodies Oscillate in Neuro 2A Cells
Source: Front Cell Neurosci. 2019 Oct 29;13:487. doi: 10.3389/fncel.2019.00487 (PMC6828937; doi:10.3389/fncel.2019.00487)
Supplement: Supplementary file 12 [file Data_Sheet_12.PDF]

Suppl. Table 9: Processing body Signal Intensity (DDX6 marker in Fig. 1).

**T (h)**	**8**	**12**	**16**	**20**	**24**	**28**	**32**	**36**	**40**	**44**	**48**	**52**	**56**	**60**	**64**	**68**
**8**																
**12**	-0.96															
**16**	37.88	38.84														
**20**	-40.33	-39.37	-78.21													
**24**	-6.50	-5.54	-44.38	33.83												
**28**	-15.98	-15.02	-53.86	24.35	-9.48											
**32**	-57.47	-56.51	-95.35	-17.14	-50.97	-41.49										
**36**	-11.69	-10.73	-49.57	28.64	-5.19	4.29	45.78									
**40**	**-150.20*** **	**-149.20*** **	**-188.10*** **	**-109.90***	**-143.70*** **	**-134.20*** **	-92.73	**-138.50*** **								
**44**	**-170.20*** **	**-169.20*** **	**-208.00*** **	**-129.80** **	**-163.70*** **	**-154.20*** **	**-112.70***	**-158.50*** **	-19.95							
**48**	**-170.80*** **	**-169.80*** **	**-208.60*** **	**-130.40*** **	**-164.30*** **	**-154.80*** **	**-113.30***	**-159.10*** **	-20.57	-0.62						
**52**	**-139.30** **	**-138.30** **	**-177.20*** **	-98.98	**-132.80** **	**-123.30***	-81.83	**-127.60** **	10.89	30.84	31.46					
**56**	**-163.10*** **	**-162.20*** **	**-201.00*** **	**-122.80** **	**-156.60*** **	**-147.10*** **	**-105.70***	**-151.40*** **	-12.93	7.02	7.64	-23.82				
**60**	**-177.90*** **	**-177.00*** **	**-215.80*** **	**-137.60*** **	**-171.40*** **	**-162.00*** **	**-120.50** **	**-166.30*** **	-27.74	-7.79	-7.17	-38.63	-14.81			
**64**	**-178.10*** **	**-177.20*** **	**-216.00*** **	**-137.80*** **	**-171.60*** **	**-162.10*** **	**-120.70** **	**-166.40*** **	-27.93	-7.98	-7.36	-38.82	-15.00	-0.19		
**68**	**-188.00*** **	**-187.10*** **	**-225.90*** **	**-147.70*** **	**-181.50*** **	**-172.10*** **	**-130.60** **	**-176.40*** **	-37.85	-17.90	-17.28	-48.74	-24.92	-10.11	-9.92	

Dunn´s Multiple Comparison test for variable Processing bodies covered by cells. Difference in rank sum.
*In bold $p \leq 0.05$.
