# Supplementary material for: Processing Bodies Oscillate in Neuro 2A Cells
Source: Front Cell Neurosci. 2019 Oct 29;13:487. doi: 10.3389/fncel.2019.00487 (PMC6828937; doi:10.3389/fncel.2019.00487)
Supplement: Supplementary file 13 [file Data_Sheet_13.PDF]

Suppl. Table 10: Processing body Area (DDX6 marker in Fig. 1).

T (h)	8	12	16	20	24	28	32	36	40	44	48	52	56	60	64	68
**8**																
**12**	-30.21															
**16**	12.34	42.55														
**20**	-45.20	-14.99	-57.54													
**24**	0.83	31.04	-11.51	46.03												
**28**	-10.88	19.33	-23.22	34.32	-11.71											
**32**	-45.08	-14.87	-57.42	0.119	-45.91	-34.20										
**36**	-4.35	25.86	-16.69	40.85	-5.18	6.53	40.73									
**40**	**-143.10**	**-112.90****	**-155.50*****	-97.91	**-143.90*****	**-132.20*****	-98.03	**-138.80*****								
**44**	**-179.00**	**-148.80*****	**-191.40*****	**-133.8****	**-179.90*****	**-168.10*****	**-133.90****	**-174.70*****	-35.91							
**48**	**-150.90**	**-120.70****	**-163.20*****	**-105.7***	**-151.70*****	**-140.00*****	**-105.80***	**-146.60*****	-7.79	28.12						
**52**	-104.00	-73.75	**-116.30***	-58.76	-104.80	-93.08	-58.88	-99.61	39.15	75.06	46.94					
**56**	**-152.80**	**-122.60****	**-165.20*****	**-107.6***	**-153.60*****	**-141.90*****	**-107.70***	**-148.50*****	-9.71	26.20	-1.92	-48.86				
**60**	**-150.00**	**-119.70****	**-162.30*****	**-104.8***	**-150.80*****	**-139.10*****	**-104.90***	**-145.60*****	-6.84	29.07	0.95	-45.99	2.87			
**64**	**-162.40**	**-132.20*****	**-174.80*****	**-117.2***	**-163.30*****	**-151.50*****	**-117.30***	**-158.10*****	-19.31	16.60	-11.52	-58.46	-9.61	-12.47		
**68**	**-175.40**	**-145.20*****	**-187.70*****	**-130.2****	**-176.20*****	**-164.50*****	**-130.30****	**-171.00*****	-32.28	3.63	-24.49	-71.43	-22.57	-25.44	-12.97	

Dunn´s Multiple Comparison test for variable Area. Difference in rank sum.
*** In bold $p \leq 0.05$.**
